# Graphene nanoplatelets induced heterogeneous bimodal structural magnesium matrix composites with enhanced mechanical properties

**DOI:** 10.1038/srep38824

**Published:** 2016-12-12

**Authors:** Shulin Xiang, Xiaojun Wang, Manoj Gupta, Kun Wu, Xiaoshi Hu, Mingyi Zheng

**Affiliations:** 1 School of Materials Science and Engineering, Harbin Institute of Technology, No. 92, West Da-Zhi Street, Harbin 150001, PR China; 2 Department of Mechanical Engineering, National University of Singapore, 9 Engineering Drive 1, Singapore 117576, Singapore

## Abstract

In this work, graphene nanoplatelets (GNPs) reinforced magnesium (Mg) matrix composites were synthesised using the multi-step dispersion route. Well-dispersed but inhomogeneously distributed GNPs were obtained in the matrix. Compared with the monolithic alloy, the nanocomposites exhibited dramatically enhanced Young’s modulus, yield strength and ultimate tensile strength and relatively high plasticity, which mainly attributed to the significant heterogeneous laminated microstructure induced by the addition of GNPs. With increasing of the concentration of GNPs, mechanical properties of the composites were gradually improved. Especially, the strengthening efficiency of all the composites exceeded 100%, which was significantly higher than that of carbon nanotubes reinforced Mg matrix composites. The grain refinement and load transfer provided by the two-dimensional and wrinkled surface structure of GNPs were the dominated strengthening mechanisms of the composites. This investigation develops a new method for incorporating GNPs in metals for fabricating high-performance composites.

In the last two decades, carbon nanotube (CNT) and graphene are two rising stars on the horizon of materials science and engineering because both of them possess high elastic modulus and high mechanical strength as well as excellent electrical and thermal conductivities. In combination with their high aspect ratio characteristics, CNTs and graphene are considered to be the most promising reinforcements for fabricating composite materials. This has prompted extensive studies to investigate the effects of carbonaceous nanomaterials as high-performance additives in different materials (polymers, ceramics and metals) to improve their mechanical properties^1^,^2^.

Graphene nanoplatelets (GNPs), or multi-layer graphene, preserving many of the appealing properties of single-layer graphene, are less expensive and easier to produce and handle^3^. Till to date, research works about carbonaceous nanomaterials reinforced metal matrix composites (MMCs) mainly concentrate on the ones reinforced with CNTs, while the research of GNPs contained MMCs is just at the beginning stage^2^. Compared with the one-dimensional carbon nanotube, the two-dimensional geometry of graphene is intrinsically more compatible in enhancing mechanical interlocking and load transfer with the matrix^4^. Despite the potential to improve the mechanical properties mentioned above, GNPs is difficult to disperse well in metals because of their large surface area which will lead to irreversible clusters and deteriorate the mechanical performance consequently. On the other hand, the majority of researches have concentrated on MMCs with a uniform distribution of reinforcement, which leads to improved stiffness and strength. Nevertheless, industrial applications of MMCs are still constrained due to their sizeable decrease of damage tolerance, such as ductility and fracture toughness^5^. One possible innovation is to attempt to design an inhomogeneous and bimodal microstructure to take advantage of the combination of ductile and strong regions, where the ductile region sustains the ductility and the strong region offers the strength^6^. Because of the sensitivity of the matrix microstructure to reinforcements, two-dimensional GNPs have the potential to realise improved microstructure of composites.

In this work, a multi-step dispersion route combining the pre-dispersing of GNPs, semi-solid stirring, high energy ultrasonic processing and hot extrusion was used for the synthesis of the GNPs induced heterogeneous bi-modal structural Mg-6Zn composites. The Mg-6Zn was selected as the matrix alloy for two reasons: (i) Mg alloys are the promising candidates to meet the soaring demand of weight critical structural applications. Mg alloys, although they have limitations (such as low elastic modulus and low strength), will be strengthened by the introduction of reinforcements with high performance and strengthening efficiency^7^; (ii) Mg-6Zn is one of the commonly used Mg alloys. The molten Mg-6Zn alloy exhibits a wide semi-solid range and promotes no chemical reactions with GNPs, making it suitable for semi-solid and liquid processing. In terms of the GNPs reinforced Mg matrix composites, Chen *et al*. have conducted the pioneering work by combining liquid state ultrasonic vibration and friction stir processing recently^8^. The uniform dispersion of GNPs, good bonding interface and increased microhardness are achieved. Their work opens a new avenue to produce GNPs reinforced Mg matrix composites and show the potential of GNPs in tuning the properties of metals. Our present research investigates the effect of the addition of GNPs on the inhomogeneous microstructure and mechanical properties of the composites. In addition, the strengthening mechanism of the composites was analysed and correlated to the evolution of microstructure. Results obtained in the present study demonstrate a much higher strengthening efficiency of GNPs as reinforcements than CNTs in Mg-based composites until now. The effective load transfer due to the two-dimensional and wrinkled surface structure of GNPs is considered to be responsible for the stiffening effect and high strengthening efficiency. This investigation also gives impetus to the development of GNPs contained MMCs with the potential for large-scale applications.

## Results and Discussion

### The dispersion and distribution of GNPs

**Figure 1:**
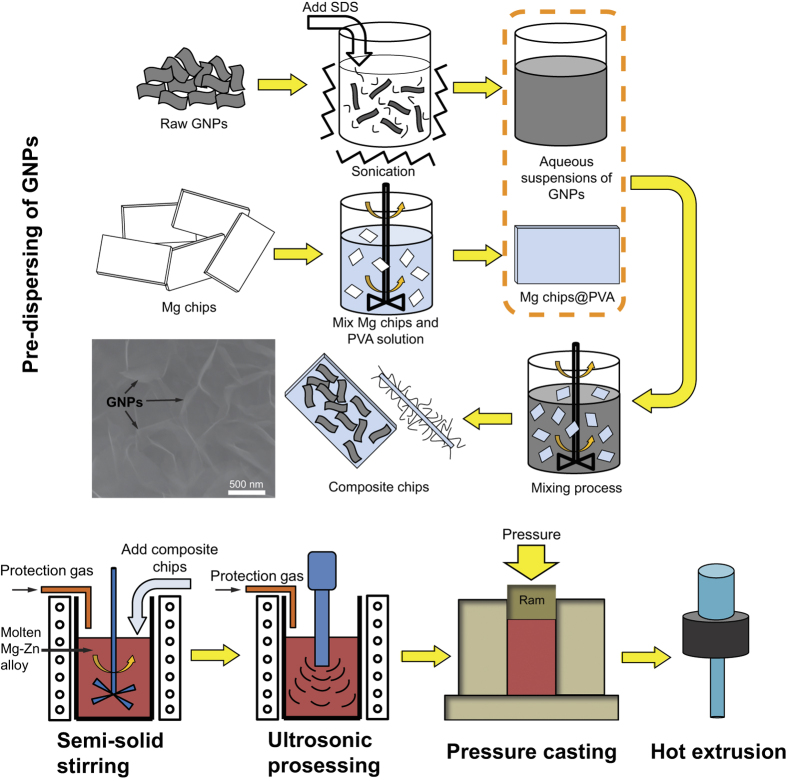
Fabrication procedure of the composites by the multi-step dispersion route. The inset SEM image shows the surface of Mg@PVA chip with absorbed GNPs.

The approach used to fabricate the composites is schematically shown in Fig. 1a and discussed in detail in the Methods. In order to eliminate the severe macro-clusters of the raw GNPs before they were mixed with the molten alloy, the aqueous process of pre-dispersing was introduced and GNPs will be kept in a repulsive condition. This is essential for the incorporation of GNPs into the melt and the dispersion of GNPs in the subsequent steps. After the pre-dispersion, individual GNPs were decorated on the Mg@PVA chip surface as shown in the inset SEM image (Fig. 1), which shows an ideal dispersion of GNPs in this step. The reason is that GNPs were converted from graphite oxide and were still partially functionalized with carboxyl groups (-COOH) at the edges due to the incomplete reduction^9^. The remaining oxygen content in the current GNPs is measured about 2.0 at.% by X-ray photoelectron spectroscopy (XPS) (see Methods and Supplementary information 1). The carboxyl groups at GNPs are stable in the molten Mg according to the chemical stability of graphite oxide below 1050 °C^10^. The PVA coated on the Mg chips provides a plenty of hydroxyl groups (–OH), which can form a stable bonding with –COOH groups^11^. Thus, Mg@PVA chips act as the carriers of GNPs to feed them into the semisolid molten alloy. The semisolid stirring prevents the severe oxidation of composite chips and the floating of GNPs because of the lower temperature and the higher viscosity of the semisolid metal than the liquid state melt, respectively^12^. In the meantime, the PVA should decompose into gas and may induce pores in the molten Mg. This was circumvented as the melt was then degassed during subsequent ultrasonic and pressure casting approaches. The homogenous dispersion of GNPs on composite chips is vital to prevent the agglomerations in the following metallurgical steps. Moreover, the later processes of semi-solid stirring, ultrasonic vibration and hot extrusion also assist in dispersion of GNPs, as reported in the previous literature^12^,^13^.

**Figure 2:**
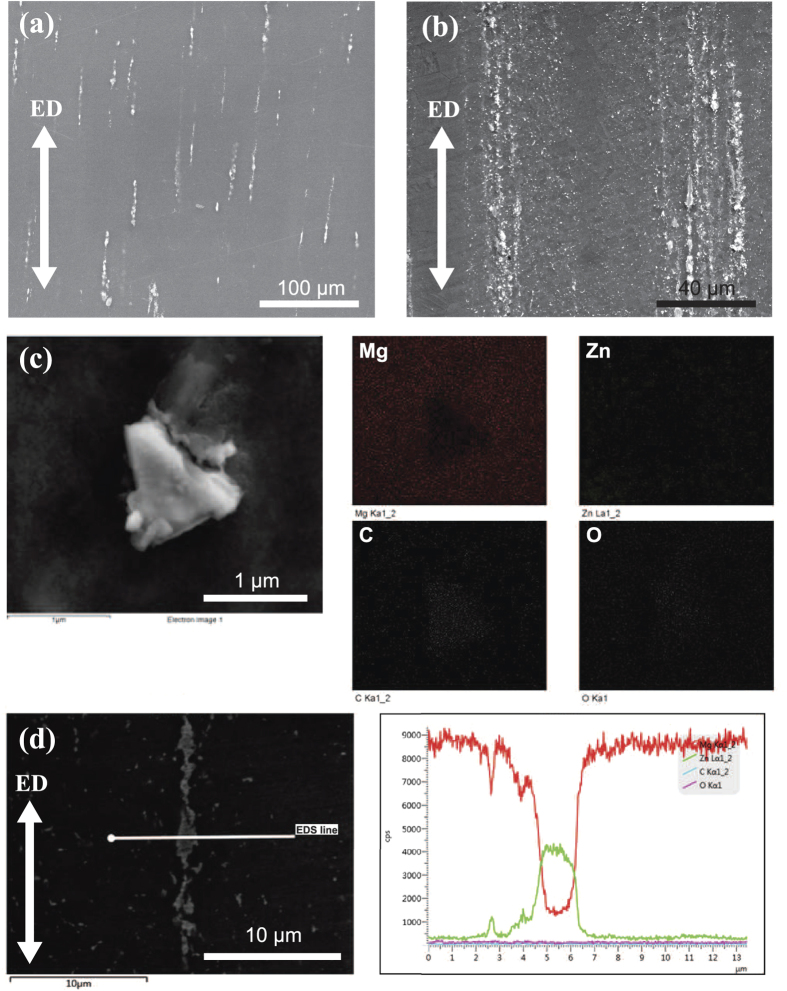
Characterization of composites. SEM images of (**a**) Mg-6Zn alloy and (**b**) 0.7 vol.% GNPs reinforced composite. (**c**) EDS map analysis of the embedded GNPs in the composite; (**d**) EDS line analysis across the extrusion band in the matrix of 0.7 vol.% GNPs reinforced composite.

Figure 2a and b are the SEM images of the as extruded Mg-6Zn alloy and the composite containing 0.7 vol.% GNPs. There are many well dispersed but inhomogeneously distributed white particle-like features in the microstructure of the composite (Fig. 2b), differing from that observed in the alloy (Fig. 2a). The EDS mapping of Mg, Zn, C and O is presented in Fig. 2c. The EDS analysis of C shows the continuous shape of the particle, which proves that GNPs exist in the composite. In both of the alloy and composites, there emerge Zn-enriched stringers parallel to the extrusion axis, as verified in the EDS line analysis in Fig. 2d. The formation of extrusion stringers is invariably associated with the concentration of shear strain in the deforming matrix^14^. Besides, both Fig. 2c and d exhibit the minor content of O on the surface, as a result of the tiny oxidation of Mg during the standard metallography preparation. However, the O area of the EDS map is brighter in the GNPs’ region than in the matrix (Fig. 2c). This higher O content is likely to have formed due to: (a) the easy adsorption of air by GNPs during the sample preparation, because of the wrinkle surface of the exposed GNPs; (b) the existence of functional groups on GNPs (see Supplementary information 1). To note here is, the distribution of GNPs in the matrix is inhomogeneous. The distribution appears as the laminated-like pattern, where the GNPs-rich region and GNPs-lean region are continuous and alternate along the direction of extrusion. The inhomogeneous distribution of GNPs in the matrix is the result of the segregation of GNPs in the cast ingot. During solidification processing, GNPs are pushed to the grain boundaries by the moving solidification front in the matrix. The subsequent hot extrusion process disintegrates the segregations of GNPs and then aligns them along the extrusion direction. Supplementary information 2 represents the evolution of the distribution of GNPs in the composite.

**Figure 3:**
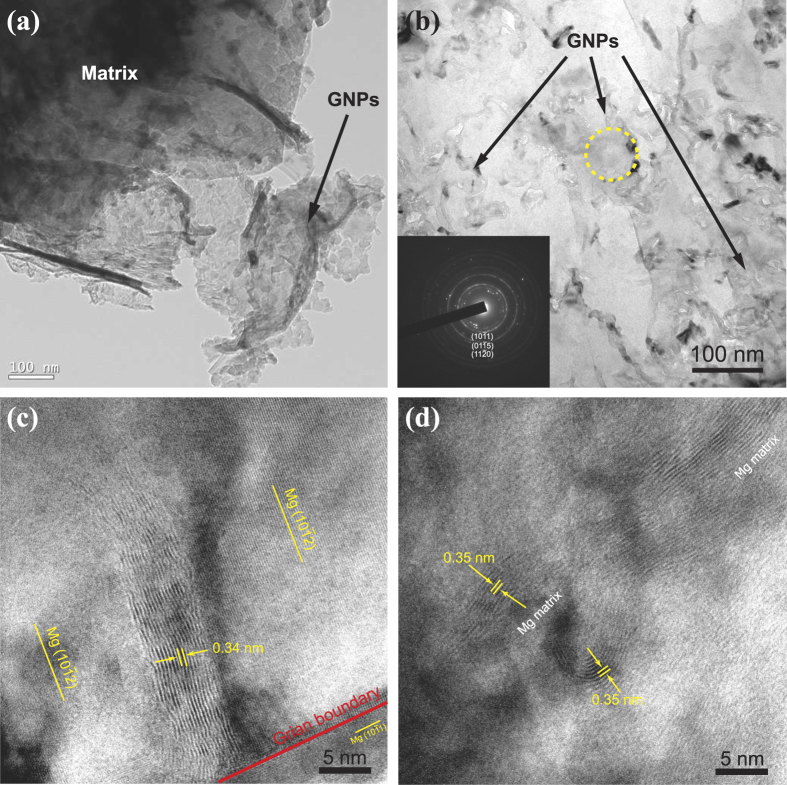
Characterization of GNPs in composites. TEM images of the wavy edge structure of GNPs in the composites with: (**a**) 1.0 vol.% and (**b**) 1.6 vol.%, the inset in (**b**) shows the diffraction pattern from the circled region. HRTEM images of GNPs embedded in the matrix of: (**c**) 1.0 vol.% composite and (**d**) 1.6 vol.% composite.

Because of the planar geometry and the matrix coating on GNPs, only the exposed edges of GNPs are discernible in SEM images. Figure 3a and b present the TEM observation and diffraction pattern of typical GNPs in the matrix, revealing a curled morphology consisting of a thin wrinkled paper-like structure. This structure suggests that the intrinsic ripples of GNPs would be helpful in promoting mechanical interlocking and load transfer with the matrix. Figure 3c and d demonstrate the side view of GNPs as well as the interface between GNPs and the matrix by high-resolution TEM (HRTEM) observation. The edges of two layers are dominated by two bright lines and no intermediate compound or pore was detected at the interface. According to an interlayer spacing of 0.34 nm for graphite, the GNP is 3–5 nm thick and composed of approximately 9–15 stacked individual monatomic graphene layers. As indicates in Fig. 3d, two pieces of GNPs are embedded in the surrounding Mg matrix, and even the extremely tiny gap between GNPs can be filled up with Mg alloy. In general, carbon (graphite) has a poor wettability by molten Mg with the contact angle of 120°^15^. The result validates that the introduction of high energy ultrasonic processing in the molten metal can immensely improve the wetting condition and overcome the attractive van der Waals force between GNPs because of the intense transient cavitation (with a temperature of about 5000 °C and pressure around 1000 atm) provided by ultrasonic wave^8^,^12^. Besides, the extrusion process would also bring about better contact between the GNPs and the matrix resulting in a stronger bond of the interface.

### Heterogeneous bimodal microstructure of the composites

**Figure 4:**
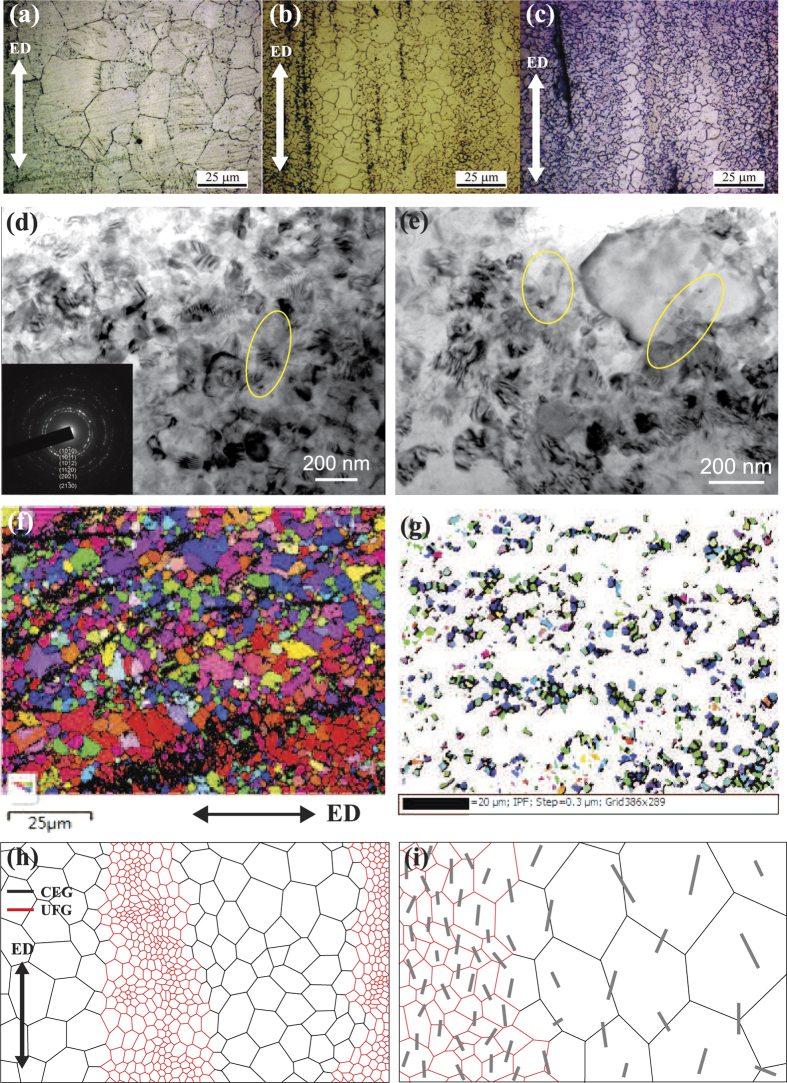
Bimodal microstructure of the composites. Optical images of (**a**) Mg-6Zn alloy, (**b**) composite with 0.7 vol.% GNPs and (**c**) composite with 1.6 vol.% GNPs. (**d**,**e**) TEM images of the grains in the UFG zone of 1.6 vol.% composite. GNPs are marked by circles. The inset in (**d**) shows the ring diffraction patterns from the polycrystalline matrix. (**f**) The EBSD image of the composite with 1.6 vol.% GNPs. (**g**) EBSD image of grains smaller than 3 μm. Schematic graphs of (**h**) the bimodal structural composite and (**i**) the correspondence of the inhomogeneous distribution of GNPs and the bimodal microstructure.

The optical microstructures of extruded alloy and composites are shown in Fig. 4a–c. The alloy has a completely recrystallized microstructure with an average grain size diameter of 20 μm. In contrast, the microstructure of the composites can be clearly divided into two types of regions according to the inhomogeneous distribution of GNPs. The grains are ultrafine in the GNPs-rich region (UFG region). Figure 4d and e present the representative TEM micrographs of the UFG in the composites with 1.6 vol% GNPs after dynamic recrystallization (DRX). Discernible GNPs are marked by yellow circles. The ring diffraction patterns (as shown in an inset in Fig. 4d) indicate that the UFG are mainly polycrystalline Mg. The grain size of UFG varies from the micron regime to the nanocrystalline range, the interference fringes are observed across some nanocrystalline grains. This indicates that these tiny grains are heavily deformed due to the pinning and obstructing by GNPs in UFG region. On the other hand, the grains in the GNPs-lean region are coarse and equiaxed (CEG region), and the grain size is about 6–10 μm. As revealed by the electron backscatter diffraction (EBSD) analysis in Fig. 4f, two zones in the composites are presented layer by layer. Figure 4g shows the distribution of grains with a size smaller than 3 μm after whiting out larger grains areas. Most UFG congregate into long lamellae along the extrusion direction, it is also apparent that the larger grains distribute within the white section in Fig. 4g. Accordingly, the schematic representation of the above bi-modal structural matrix is shown in Fig. 4h.

The heterogeneous microstructure of composites and the refined grains in both CEG and UFG zones prove that GNPs significantly affect the DRX of Mg matrix. In general, the scale of the DRX grain size depends on both nucleation and grain growth. Unlike the DRX behaviour in pure alloys, DRX nucleation in nanocomposites can be stimulated by the presence of GNPs and also grain growth will be obstructed concurrently, leading to the refined grains in both CEG and UFG zones. The sensitivity of recrystallization to local fraction of GNPs gives rise to the substantially smaller grains in the GNPs-rich region (UFG region). The correspondence of the inhomogeneous distribution of GNPs and the bimodal microstructure of the composite is illustrated in Fig. 4i. By comparing Fig. 4b and c, as the content of GNPs increases, a higher proportion of UFG zones are obtained in the composites. This morphology of lamellar microstructure is advantageous in improving the strength and sustaining the ductility of composites, which will be discussed in the next section.

### Mechanical properties

**Figure 5:**
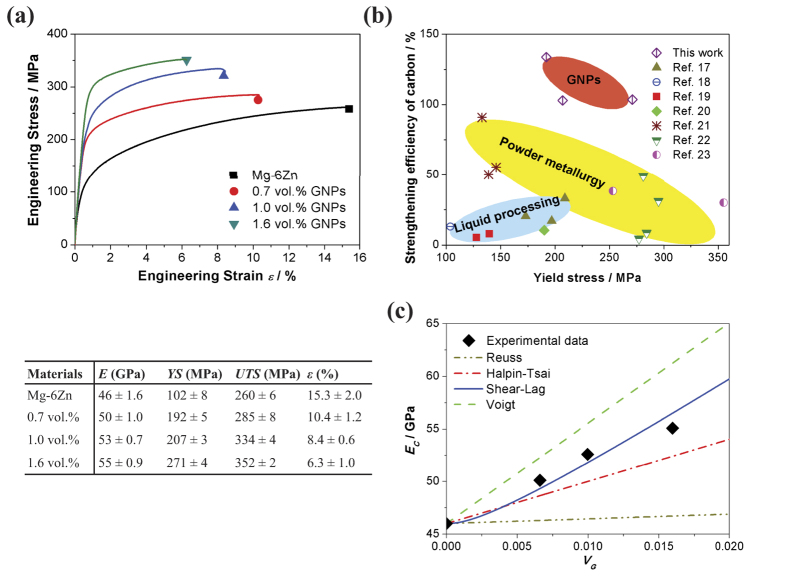
Mechanical properties of the composites. (**a**) Tensile stress–strain curves of as-extruded alloy and composites. The inset table shows the tensile properties. (**b**) Yield strength versus strengthening efficiency of GNPs/Mg-6Zn in this work in comparison with results from CNTs reinforced Mg-based MMCs. (**c**) The variation in *E*_c_ as a function of *V*_G_ for different micromechanical models (Voigt-Reuss, Halpin-Tsai and Shear-lag), compared with the present experiment result.

The typical tensile stress-strain curves of composites and the unreinforced alloy are shown in Fig. 5a. The values of Young’s modulus (*E*), yield stress (*YS*), ultimate tensile strength (*UTS*), and elongation (*ε*) from the engineering stress-engineering strain curves are provided in the inset table. The as-extruded composites exhibited a simultaneous increment in Young’s modulus, *YS* and *UTS* with an increasing concentration of GNPs. The Young’s modulus, *YS* and *UTS* of 55 GPa, 271 MPa and 352 MPa were realised in the 1.6 vol.% composite, which were improved by 20%, 166% and 35%, respectively, compared with that of the unreinforced alloy. The strengthening efficiency is generally used to characterise the strengthening effect of reinforcement. The strengthening efficiency of yield strength was determined by *R* = (*σ*_*yc*_ − *σ*_*ym*_)/(*V*_*G*_*σ*_*ym*_)^16^. The calculated data is also compared with the experimental results of CNTs contained Mg matrix composites in the open publications^17^,^18^,^19^,^20^,^21^,^22^,^23^. The GNPs show the strengthening efficiency of more than 100%, whereas that of CNTs rarely exceeds 100% by various preparation methods, as shown in Fig. 5b. The higher strengthening efficiency makes GNPs a promising carbonaceous reinforcement in the new class of high-performance MMCs.

Elongation of metals relates closely to strain hardening. The reduced ductility should be caused by four factors: (1) The nano-sized grains in UFG zones, which tend to lose the strain hardening ability quickly on deformation owing to their very low dislocation storage efficiency inside^24^. (2) Agglomerations of GNPs tend to favour the formation of voids. (3) GNPs located on grain boundaries can lead to defects in the matrix that act as seed points for crack initiation and premature fracture. (4) Stress concentration in the vicinity of GNPs is also detrimental to the ductility. Compared to the CEG zone, GNPs have a higher concentration in the local UFG zone. As a consequence, the increase in the fraction of UFG zone gives rise to the deterioration in ductility. However, the loss in ductility can be improved by the strain hardening provided by dislocation activity in the larger CEG zones in the matrix. When under tensile stress, dislocation sources in the CEG lamellae are activated first and such grains are prone to plastic deformation. However, the CEG zones are constrained by the stronger UFG zones. Therefore, dislocations pile-up at grain boundaries of CEG cannot glide easily. The pile-ups consequently generate a long range back stress that acts on the obstacle, preventing further dislocation motion so that the flow stress must rise to continue to operate the source of dislocations at a higher strain^25^. In this way, for the composites with the same low content of reinforcement, a bimodal grain size distribution is a possible means to combine ideal strength and ductility compared to the uniformly refined grain composites: the UFG regime helps enhance the strength to a large extent, meanwhile, the CEG regime with sufficient large size accommodates the useful uniform deformation to useful strains.

### Stiffening and strengthening mechanisms of composites

Figure 5c shows the calculated Young’s modulus of composites plotted as a function of *V*_*G*_ by some of the most commonly used models (i.e. Voigt-Reuss model^14^, Halpin-Tsai model^26^ and Shear-lag model). The experimental results are also marked in the chart. In the shear-lag approach and Halpin-Tsai equation, one piece of GNP is modelled as a square cross-section platelet having length/width and thickness (*t*). As seen in the SEM and HRTEM images (see Supplementary Fig. S1 and Fig. 3), the length/width along the tensile direction and thickness of GNPs is about 1–2 μm and 5 nm, respectively. From the graphical representation, the shear-lag approach matches the experiment data better. The stress transfer from the matrix to GNPs is assumed to take place through an interfacial shear stress^27^. That is, the modulus of the composite can be calculated from the equation (see Supplementary information 3):


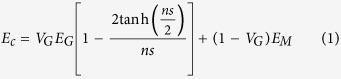


where


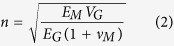


and *E*_*G*_ and *E*_*M*_ are the Young’s modulus of the GNPs (~1 TPa) and the matrix, respectively, *s* is the aspect ratio of GNP (*d*_*G*_*/t*), *d*_*G*_ is the distance in the direction of tensile axis across the GNPs and *V*_*G*_ is the volume fraction of GNPs. The under-prediction of the theoretical calculation (0.7 and 1.0 vol.% GNPs) could be a consequence of the wrinkled surface of GNPs, which have the larger surface area than the platelets with flat surface assumed by the shear-lag model. When the content of GNPs increases to 1.6 vol.%, more micro-clusters of GNPs emerges in the composites. As a result, the calculated value exceeds the experimental result (Fig. 5c).

In the present work, the yield strength of composites increases with the addition of GNPs. Several possible factors can contribute to this: (i) grain size refinement (*Δσ*_*gr*_); (ii) load-transfer effects due to the presence of GNPs (*Δσ*_*lt*_); (iii) generation of the dislocation density due to strain generated by the thermal expansion mismatch between the matrix and GNPs (*Δσ*_*tm*_); (iv) Orowan strengthening caused by the resistance of closely spaced GNPs to the passing of dislocations (*Δσ*_*or*_). Then the predicted overall yield strength for the composites can be estimated as^28^


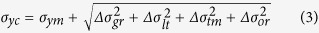


where, *σ*_*ym*_ stands for the yield strength of the Mg-6Zn matrix.

The microhardness results of CEG and UFG zones of the composites, as well as the alloy, are shown in Supplementary Fig. S4. It is notable that the hardness values of the CEG and UFG zones are similar in composites with different GNPs contents. This indicates the CEG and UFG zones in different composites exhibit similar grain size and density of GNPs. In other words, the content of GNPs for the whole sample did not evidently affect the grain size and GNPs density in CEG and UFG zones, respectively, but the content of GNPs changed the volume fractions of these two local zones in the bimodal microstructure (see Fig. 4). It is no longer available to use the concept of average grain size to estimate *Δσ*_*gr*_. The refined grains of both the CEG and UFG lamellae contribute to the yield strength of the composites in conjunction, as can be calculated by the Hall-Petch equation, independently^17^:


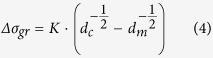


where *K* is the is a constant (280 MPa∙μm^1/2^)^28^ and *d*_*c*_ and *d*_*m*_ are the average grain size of composites (CEG or UFG zone) and the matrix without GNPs. From the Hall-Petch equation, the calculated yield strengths increment for the CEG and UFG lamellae are 33.5 MPa and 115 MPa, respectively. According to the rule of mixtures, the total strength contribution from grain boundaries will range between these two values in terms of different fractions of these two local zones. Calculation of the fractions of two lamellae in the composites is made by measuring the area of each zone in the low magnification optical microscopy images.

Using the modified shear-lag model^29^, the relationship between *σ*_*yc*_ and *σ*_*ym*_ based on the load transfer mechanism is derived as follows,


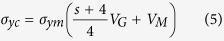


where *V*_*m*_ is the volume fraction of the matrix and *V*_*G*_ + *V*_*m*_ = 1. The increment in *YS* due to the load transfer effect can be calculated as follows:


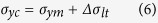


by substituting Eq. (6) into Eq. (5) yields,


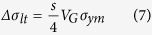


**Figure 6:**
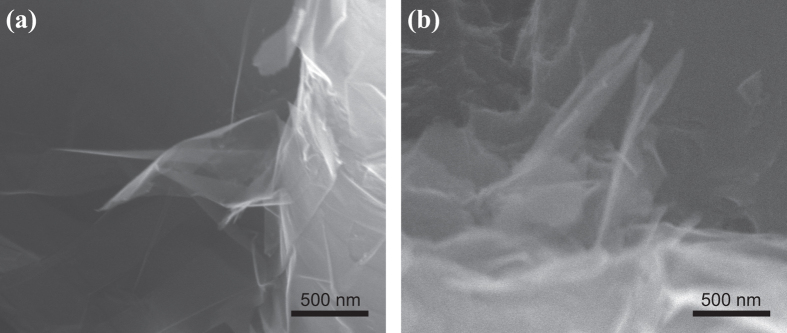
SEM fractographs of the composites with: (**a**) 0.7 vol.% GNPs and (**b**) 1.0 vol.% GNPs.

**Figure 7:**
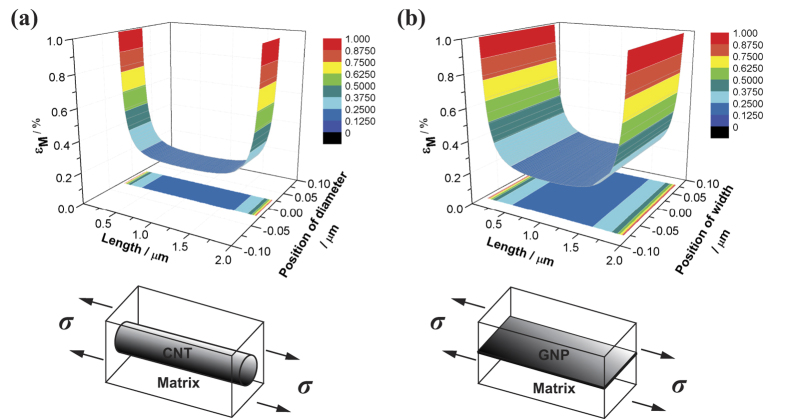
The calculated comparison of the strain distributions and the projections in the matrix when the strain of the reinforcement is 0.2%. (**a**) A CNT is the reinforcement, assuming that the size of CNT is 2 μm in length, 0.05 μm in diameter and 5 nm in thickness, respectively. (**b**) An “unzipped GNP from the CNT” is the reinforcement, which possesses the size of 2 μm in length, 0.05π μm in width and 5 nm in thickness, respectively.

Compared to CNTs, there are two main advantages for GNPs in load transfer effect. First, there is a larger surface area on GNPs available for interaction with the matrix. Since both surfaces of each individual GNP will contact the matrix, rather than only the outer surface of CNTs. Figure 6a and b show two representative SEM micrographs taken from the tensile fracture surfaces, which indicate GNPs sticking out of the fracture matrix. The wrinkled edges of GNPs at the nano-scale can be distinguished clearly. The persistent wavy structure of GNPs within the composite provides for better interaction with the host Mg matrix. The micrographs also suggest that GNPs in the matrix retain extremely small thickness; otherwise, GNPs will turn to straight platelets when the thickness increases gradually due to the out-of-plane rigidity^30^. However, the pulled out GNPs in the fracture surface do not indicate the weak interface between GNPs and the matrix. Whether pulling-out or pulling-off of the fibre is strongly dependent on the critical length (*l*_*c*_) which was defined by Kelly and Tyson^2^. For the length of fibre less than *l*_*c*_, the fibre would experience no fracture while the composite fails. As a result, relatively larger GNPs will be favourable for improving the strengthening effect and efficiency. Second, when the composites are subjected to axial stress, dislocations are accumulated or tangled during plastic deformation, leading to plastic strain fields near GNPs. It is similar to the plastic deformation behaviour of the CNTs/aluminium composites^31^. From the calculation of shear-lag model (Supplementary information 3 and ref. 14), Fig. 7 represents the comparison of the strain fields in the metal matrix (*ε*_*M*_) reinforced by a CNT and an “unzipped GNP from the CNT”, assuming that the strain of the reinforcement is 0.2%. For the CNT and GNP with the same amount of carbon, the two-dimensional structure of the GNP provides a wider edge area than the CNT, so the larger plastic strain fields can be developed near the edges of GNP. These larger plastic strain fields may hold higher applied stress which corresponds to the higher load transfer efficiency. Hence, GNPs display a higher strengthening efficiency than CNTs.

For *Δσ*_*tm*_, which is induced by the residual plastic strain developed due to the difference in the coefficients of thermal expansion (CTE) between the matrix and the GNPs during the post-fabrication cooling is given by^32^:


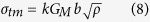


where, *k* is a constant, calculated to be 1.25, *b* is the Burgers vector of the matrix, *G*_*M*_ is the shear modulus of the matrix, *ρ* is the detailed derivation for the increase in geometrically necessary dislocations, can be obtained from ref. 14,


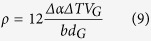


where, *Δα* is the difference in CTE between the matrix and GNP (*Δα* = *α*_*M*_ − *α*_*GNP*_), *ΔT* is the difference between the extrusion and test temperatures, *d*_*G*_ is the size of a GNP. The data of GNPs/Mg-6Zn composites used are: *G*_*M*_ = 17.7 GPa, *b* = 0.32 nm, *α*_*M*_ = 28.4 × 10^−6^ (°C)^−1^ 33, *α*_*GNP*_ = −8 × 10^−6^ °C)^−1^ 34, *ΔT* = 280 °C, *d*_*G*_ = 1.5 μm. The increment in *YS* due to Orowan strengthening can be described by the Orowan-Ashby equation^35^:


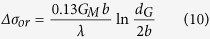


where, *λ* is the mean interparticle distance given by:


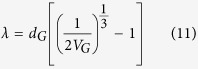


**Figure 8:**
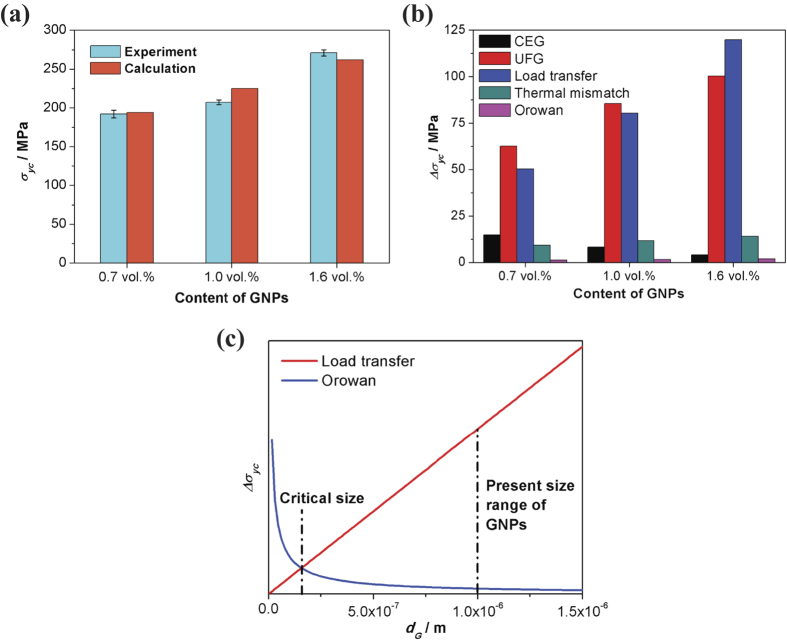
Theoretical calculation of the strengthening mechanisms. (**a**) The *σ*_*yc*_ comparison between calculation and experiment values. Error bars represent s.d. of three data sets. (**b**) Yield strength increment according to different strengthening mechanisms, the grain size strengthening is divided into the effect of CEG and UFG, respectively. (**c**) Relationship between load transfer and Orowan strengthening effect for the yield strength of the composites with the increasing *d*_G_ of GNPs.

The theoretical value of the yield strength of the composites and the quantification of different strengthening mechanisms are shown in Fig. 8a and b. The calculated *σ*_*yc*_ by Eq. 3 are in good agreement with the experimental results (Fig. 8a). According to the column chart in Fig. 8b, the load transfer effect has surpassed the fine-grained effect and become the main strengthening mechanism as the content of GNPs is increasing. Compared to other particle and fibre type reinforcements, the GNP has a huge aspect ratio as the two-dimensional and wavy structure, which can provide a huge surface area available for interaction with the matrix. It is noteworthy that the Orowan mechanism takes the least effect on strengthening in the calculation. However, Orowan strengthening caused by the resistance of closely spaced hard particles to the passing of dislocations is important in many metal matrix composites^35^. The reason for this insignificance is because the diameter of GNPs is coarse (*d*_*G*_ = 1–2 μm) so the inter-particle spacing is large when compared with other nano-size particle reinforcements. Owing to the two-dimensional structure of GNPs, load transfer and Orowan strengthening can never be enhanced concurrently. This explanation is clearly proven in Fig. 8c. We assume that the thickness and the amount of GNPs are constant, so these two strengthening factors of composites are the univariate functions of GNP’s size (*d*_*G*_) only. The chart displays that if only *d*_*G*_ is lower than the critical size (about 150 nm) can Orowan strengthening exceed the effect of load transfer, and vice versa. This conclusion is also well verified by Kim *et al*.^36^, where the nano-sized particles of GNPs (*d*_*G*_ = 5–15 nm) in the composites make Orowan strengthening as the major strengthening mechanism. Nevertheless, on condition that even though the GNPs flakes can be fully refined to nano-size particles in the matrix, the effect of Orowan strengthening can be overestimated. This is because that: (i) a portion of GNPs are likely to lie on grain boundaries (see Fig. 4), which are not expected to effectively impede the movement of dislocations in grain interiors; (ii) GNPs are so narrow that they can be creased in the deforming procedure of composites, leading to a lessening of their effectiveness as obstacles to dislocation motion. On the other hand, in order to achieve the GNPs flakes into nano-size particles, a high energy mechanical milling process must be added in the fabrication, however, this process is not beneficial for reducing the cost of composites from the practical prospect.

In summary, the GNPs reinforced Mg-6Zn composites with heterogeneous bimodal microstructure were fabricated by the multi-step dispersion method. All the composites exhibit significantly enhanced Young’s modulus, yield strength and ultimate tensile strength as well as the acceptable elongation compared to unreinforced Mg-6Zn. The strengthening mechanism of the GNPs/Mg-6Zn composites mainly stems from the refinement in grain size and the load transfer by the addition of GNPs. Besides, the shear-lag model is proposed to describe the high load transfer efficiency of GNPs. On account of the two-dimensional structure and the wavy surface texture compared with CNTs, GNPs are the promising reinforcements to obtain high-stiffness and high-strength MMCs. The current research shows that the combination of GNPs and the developed liquid metallurgy equipment has the advantage to produce large quantities of bulk GNPs/metal composites with enhanced mechanical properties and tailored microstructure. Future comprehensive studies are needed to explore and exploit the two-dimensional effect of GNPs in the composites thoroughly.

## Methods

### Raw materials

The GNPs were purchased from Knano Co. Ltd. (P.R. China) produced by the thermal reduction of graphite oxide. As shown in Supplementary Fig. S1b, raw GNPs are agglomerated powders with fluffy structure, which exhibit the irregular-shaped flakes morphology with mean diameters ranging from 1–3 μm. The thickness of GNPs is less than 10 nm according to the data sheet, which corresponds to approximately 1–30 sheets of graphene (assuming that the thickness of monolayer graphene is 0.34 nm). The pure Mg ingot and pure Zn ingot were obtained from Yueyang Aerospace New Materials Co. Ltd. (P.R. China). Mg chips were machined from Mg ingot, as shown in Supplementary Fig. S1c.

### Fabrication of the composites

To disperse the GNPs effectively into the Mg alloy matrix, the multi-step dispersion method combining pre-dispersing of GNPs, semi-solid stirring, high energy ultrasonic processing and hot extrusion was developed. In this study, composites with different contents of GNPs (0.7, 1.0 and 1.6 vol.%) were fabricated by this process.

First, an aqueous suspension of GNPs stabilised with an anionic surfactant (sodium dodecyl sulphate, SDS) was prepared under ultrasonic exfoliation at a power of 60 W. Concentration of GNPs up to 5 mg/mL was achieved through the continuous addition of SDS gradually during the ultrasonic exfoliation process^37^. The concentration of SDS in water solution was restricted to 1.5 mg/mL. The content of SDS is extremely low, thus the influence on the microstructure and properties of the composites can be neglected.

Mg chips were then added to the 3 wt.% polyvinyl alcohol (PVA) aqueous solution and stirred for 1 h, filtered and then rinsed with deionized water to obtain the PVA-modified Mg (Mg@PVA) chips. After adding Mg@PVA chips into the aqueous suspension of GNPs, the mixture was further stirred at 250 rpm inside a fume cupboard by heating to evaporate off the water. After that, Mg@PVA chips coated with GNPs (GNPs/Mg@PVA) were obtained. The fraction of GNPs on the chip surface could be controlled by changing the weight ratio of chips and the aqueous suspension of GNPs. Importantly, the temperature of evaporation must be controlled about 35 °C to prevent the notable oxidation of Mg. The extent of oxidation during the aqueous processing can be computed from the content of oxygen in the final composites, as seen in Supplementary information 5.

Next, Mg ingot was melted at 700 °C in CO_2_ and SF_6_ protective atmosphere, and then Zn ingot was added into the crucible. After the Zn was melted, the system was cooled to 600 °C at which the matrix alloy is in semi-solid condition. As the GNPs/Mg@PVA chips were quickly added into the semi-solid alloy, the melt was stirred in the protective atmosphere of CO_2_ and SF_6_ continuously to avoid burning. PVA should be removed because it converts to gaseous form at a temperature over 600 °C. The stirring rate was in the range of 800–1200 r/min and the stirring time was 10 min. After the semi-solid stirring step, the mixture of the melt and GNPs was rapidly reheated to 700 °C, and then the melt was ultrasonically processed at 500 W power level for 20 min before the ultrasonic probe was removed from the slurry. After the ultrasonic treatment, the slurry of GNPs and the matrix alloy was poured into a preheated steel mould (375 °C) and allowed to solidify under 100 MPa pressure to obtain the composite ingots without porosity.

After the liquid metallurgy steps, the cast billet was cut into the samples with the size of Ø 60 mm × 50 mm and homogenized at 340 °C for 12 h, then the composite was hot extruded at 300 °C with an extrusion ratio of 14:1, using a press with a 2000 kN load limit. In order to identify the effects of GNPs on the matrix alloy, the monolithic Mg-6Zn alloy was also fabricated using the same experimental parameters.

### Analysis of composition, microstructure and mechanical properties

The composition of the materials was determined using energy dispersive spectroscopy (EDS) (INCAx-act, Oxford Instruments, UK) and X-ray photoelectron spectroscopy (XPS) (ESCALAB 250Xi, Thermo Fisher Scientific, USA). The optical microscopy (OM) (P-3, Olympus, Japan), field-emission scanning electron microscopy (SEM) (Quanta 200FEG and FEI Co. Ltd., USA) and transmission electron microscopy (TEM) (Tecnai G2 F30, USA) were used to study the microstructure of the matrix and the composite. The specimens for microstructural analysis were prepared by the conventional mechanical grinding, polishing and etching in the acetic-picral solution (4.2 g of picric acid, 10 ml of acetic acid, 70 ml of ethanol and 10 ml of water) for 5–10 s. The specimens for TEM investigation were prepared by grinding-polishing to produce a foil of 50 μm thickness, followed by ion beam thinning. The electron backscatter diffraction (EBSD) analysis was conducted under a voltage of 12 kV in Oxford Instruments Aztec 2.0 system to evaluate the detailed microstructure of the composites. The cross-sectional sample for EBSD was mechanically ground carefully followed by electrolytic polishing in the electrolyte of 37.5 vol.% phosphoric acid and 62.5 vol.% ethanol at 0.3–0.5 A at room temperature for 30–60 s. Tensile testing was carried out at a crosshead speed of 0.5 mm/min using Instron-1186 tension machine. The tensile was carried out in accordance with ASTM: E8/E8M-13a standard. For each material, three samples with standard dog-bone shape were tested. Vickers hardness measurements were made for different lamellae in the matrix under loads of 245.2 mN with a dwelling time of 15 s, using Shimadzu HMV automatic digital microhardness tester.

## Additional Information

**How to cite this article:** Xiang, S. *et al*. Graphene nanoplatelets induced heterogeneous bimodal structural magnesium matrix composites with enhanced mechanical properties. *Sci. Rep.* **6**, 38824; doi: 10.1038/srep38824 (2016).

**Publisher's note:** Springer Nature remains neutral with regard to jurisdictional claims in published maps and institutional affiliations.

## Supplementary Material

Supplementary Information
