# Supplementary material for: Graphene nanoplatelets induced heterogeneous bimodal structural magnesium matrix composites with enhanced mechanical properties
Source: Sci Rep. 2016 Dec 12;6:38824. doi: 10.1038/srep38824 (PMC5150526; doi:10.1038/srep38824)
Supplement: Supplementary Information [file srep38824-s1.pdf]

Graphene nanoplatelets induced heterogeneous bimodal structural magnesium matrix composites with enhanced mechanical properties

Shulin Xiang [a, b], Xiaojun Wang [a, *], Manoj Gupta [b], Kun Wu [a], Xiaoshi Hu [a], Mingyi Zheng [a]

[a] *School of Materials Science and Engineering, Harbin Institute of Technology, No. 92, West Da-Zhi Street, Harbin 150001, PR China*
[b] *Department of Mechanical Engineering, National University of Singapore, 9 Engineering Drive 1, Singapore 117576, Singapore*

**Corresponding author. Tel: +86 45186402291. E-mail: xjwang@hit.edu.cn (Xiaojun Wang)*

Supplementary Information:

Supplementary information 1

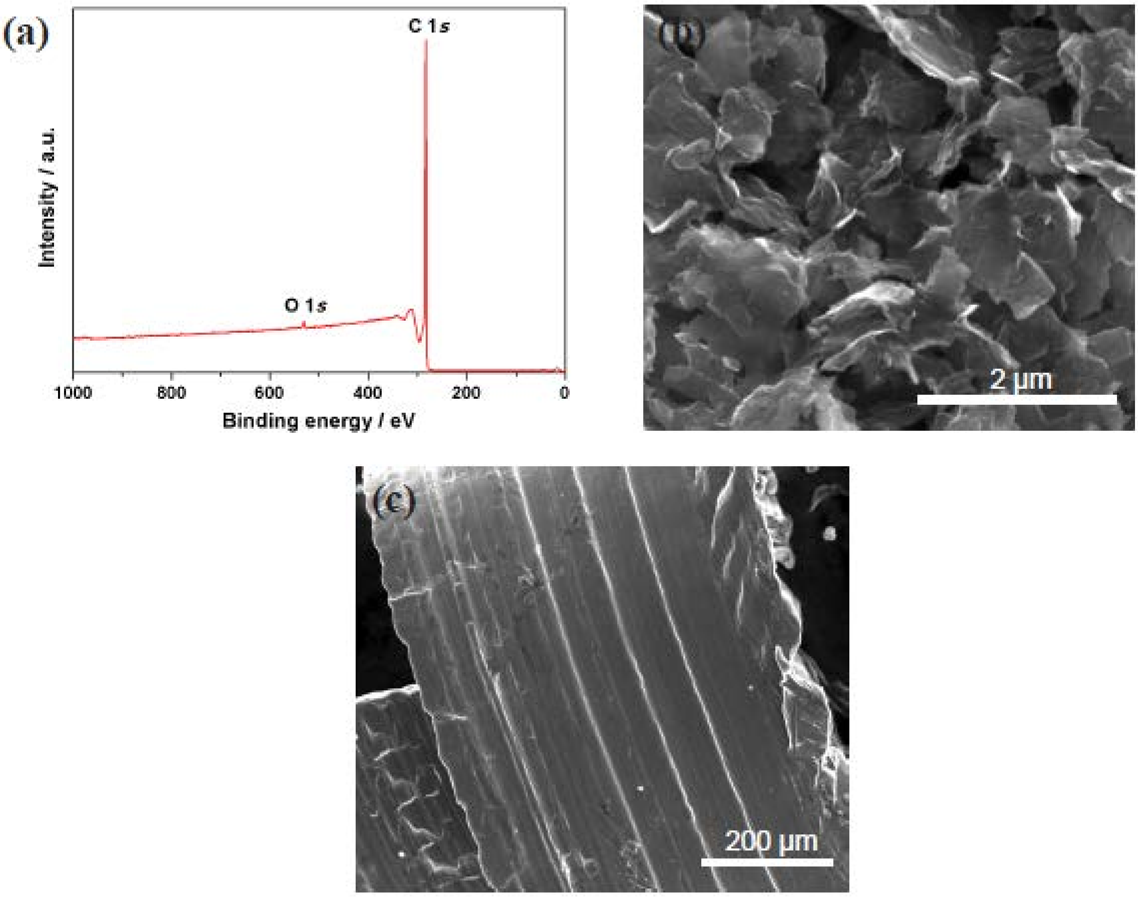

Figure S1. (**a**) XPS survey spectrum of the GNPs. The morphologies of (**b**) raw GNPs and (**c**) Mg chips.

Supplementary information 2

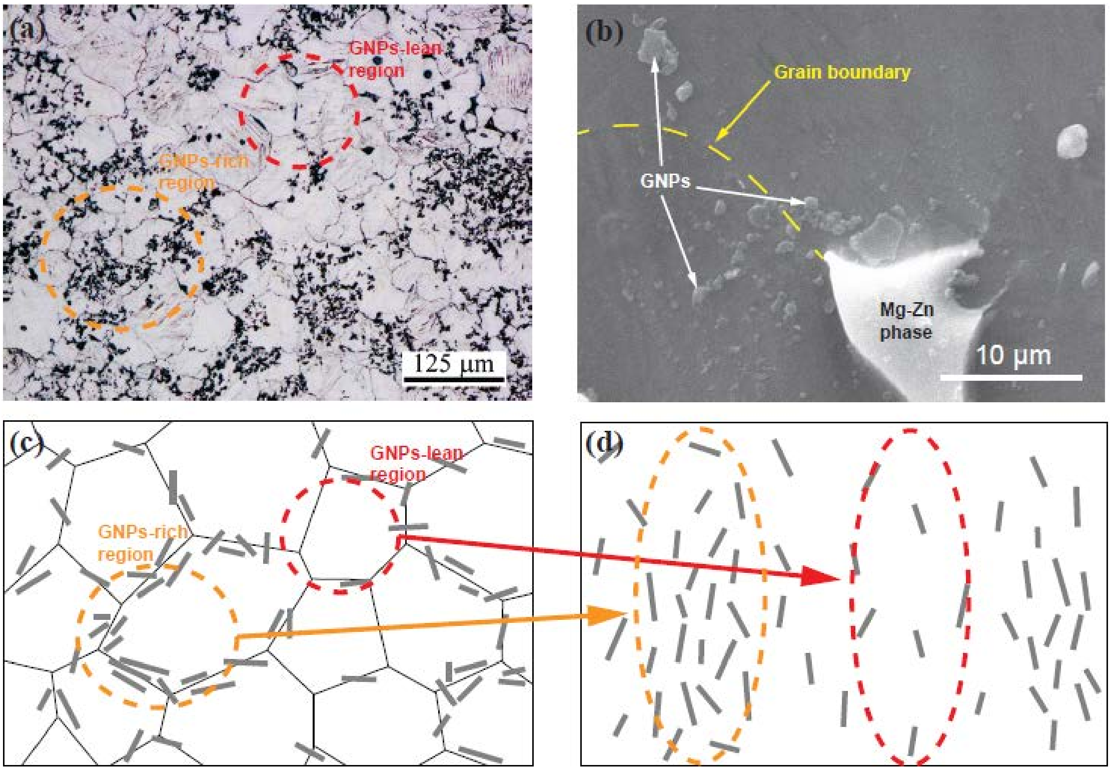

Figure S2. Microstructure of the as-cast composite. (**a**) The OM image of 1.6 vol.% GNPs reinforced composite, GNPs-rich and lean regions are labeled. (**b**) The SEM image of the segregation of GNPs in the vicinity of the grain boundary. **Schematics represent the formation of the inhomogeneous distribution of GNPs in the composite.** (**c**) the distribution of GNPs in the as-cast composite and (**d**) the alignment of GNPs in the composite after hot extrusion.

Supplementary information 3

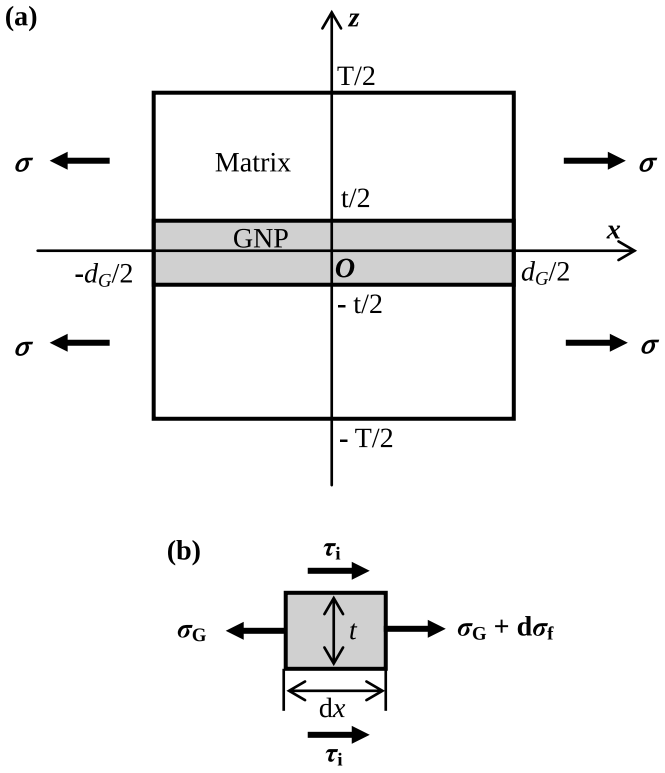

Figure S3. (**a**) Schematic illustration of the GNP within the matrix in the shear-lag model; (**b**) Balance of stresses acting on an element of length d*x* in the GNP.

According to the shear-lag model, in the system of discontinuous GNPs reinforced composites, the transfer of tensile stress from the matrix to GNPs takes place by means of interfacial shear stresses, as shown in Fig. S3a and b. The variation of tensile stress dσ_G in the GNP of a given thickness *t*, is obtained by equating the interfacial shear stress (τ_i) with length d*x*,

$$\tau_i dx = -t d\sigma_G$$

$$\frac{d\sigma_G}{dx} = -\frac{\tau_i}{t}$$

In the stage of elastic deformation, we assume that the GNP-matrix interface remains intact. We introduce *u* as the displacement of the matrix in the x direction, the increment of this displacement, d*u*, on moving out from the *z*-axis (as seen in Fig. S3a), is then determined by the shear strain *γ*, and hence by the shear modulus G_M,

$$\frac{du}{dz}=\gamma=\frac{\tau_i}{G_M}$$

for a given z, the difference between the displacement of the matrix at z = T/2 and that of the interface (z = t/2) can be expressed as:

$$\int_{u_{t/2}}^{u_{T/2}} du=\frac{\tau_i}{G_M}\int_{t/2}^{T/2} dz$$

$$u_{T/2}-u_{t/2}=\frac{\tau_i}{2G_M}(T-t)=-t\cdot\frac{d\sigma_G}{dx}\cdot\frac{T-t}{2G_M}=\frac{-t(T-t)}{2G_M}\cdot\frac{d\sigma_G}{dx}$$

the strain of GNP, ε_G and the strain of matrix, ε_M can be approximated as $\varepsilon_G \approx du_{t/2}/dx$ and $\varepsilon_M \approx du_{T/2}/dx$,

$$\frac{d(u_{T/2}-u_{t/2})}{dx}=\varepsilon_M-\varepsilon_G=\frac{-t(T-t)}{2G_M}\cdot\frac{d^2\sigma_G}{dx^2}$$

It should be noted that this assumption is not rigorous, but it is expected to represent a fairly good and simple illustration of the process of stress transfer from the matrix to GNP. Consider that T >> t,

$$\varepsilon_G-\varepsilon_M=\frac{tT}{2G_M}\cdot\frac{d^2\sigma_G}{dx^2}$$

$$\frac{d^2\sigma_G}{dx^2}=\frac{n^2}{t^2}(\sigma_G-\varepsilon_M E_G)$$

where $n=\sqrt{\frac{2G_M V_G}{E_G}}=\sqrt{\frac{E_M V_G}{E_G(1+\nu_M)}}$.

This standard second order linear differential equation has the general solution

$$\sigma_G=E_G\varepsilon_M+A\cdot\sinh\left(\frac{nx}{t}\right)+B\cdot\cosh\left(\frac{nx}{t}\right)$$

The boundary conditions are that σ_G = 0 at x = ± d_G/2, d_G is the distance in the direction of tensile axis across the GNP. This gives the solution as:

$$\sigma_G=E_G\varepsilon_M\left[1-\frac{\cosh\left(\frac{nx}{t}\right)}{\cosh\left(\frac{nd_G}{2t}\right)}\right]$$

so the strain distribution of the matrix is given by:

$$\varepsilon_M = \varepsilon_G \Bigg/ \left[1 - \frac{\cosh\left(\frac{nx}{t}\right)}{\cosh\left(\frac{nd_G}{2t}\right)}\right]$$

For the composites, the applied load can be expressed in terms of the contributions from the components according to “rule of averages”,

$$\sigma_C = V_G \sigma_G^A + (1 - V_G)\sigma_M^A$$

where, σ_G^A and σ_M^A are the average volume stresses carried by GNP and matrix

$$\sigma_G^A = \frac{1}{d_G}\int_{-d_G/2}^{d_G/2} E_G \varepsilon_M \left[1 - \frac{\cosh\left(\frac{nx}{t}\right)}{\cosh\left(\frac{nd_G}{2t}\right)}\right] dx = E_G \varepsilon_M \left[1 - \frac{2\tanh\left(\frac{ns}{2}\right)}{ns}\right]$$

in which s is the aspect ratio of the GNP (d_G/t). For the matrix, it is conventional to turn to the presumption of a uniform tensile strain, ε_M equal to that of the composite, ε_C. Finally, the Young’s modulus of the composites can be given as:

$$E_C = \frac{\sigma_C}{\varepsilon_C} = \frac{V_G \sigma_G^A + (1 - V)\sigma_M^A}{\varepsilon_M} = V_G E_G \left[1 - \frac{2\tanh\left(\frac{ns}{2}\right)}{ns}\right] + (1 - V_G)E_M \,.$$

References

[1] Clyne TW, Withers PJ. An introduction to metal matrix composites. Cambridge: Cambridge University Press; 1993.

[2] Gong L, Kinloch IA, Young RJ, Riaz I, Jalil R, Novoselov KS. Interfacial Stress Transfer in a Graphene Monolayer Nanocomposite. Adv Mater. 2010;22(24):2694-7.

Supplementary information 4

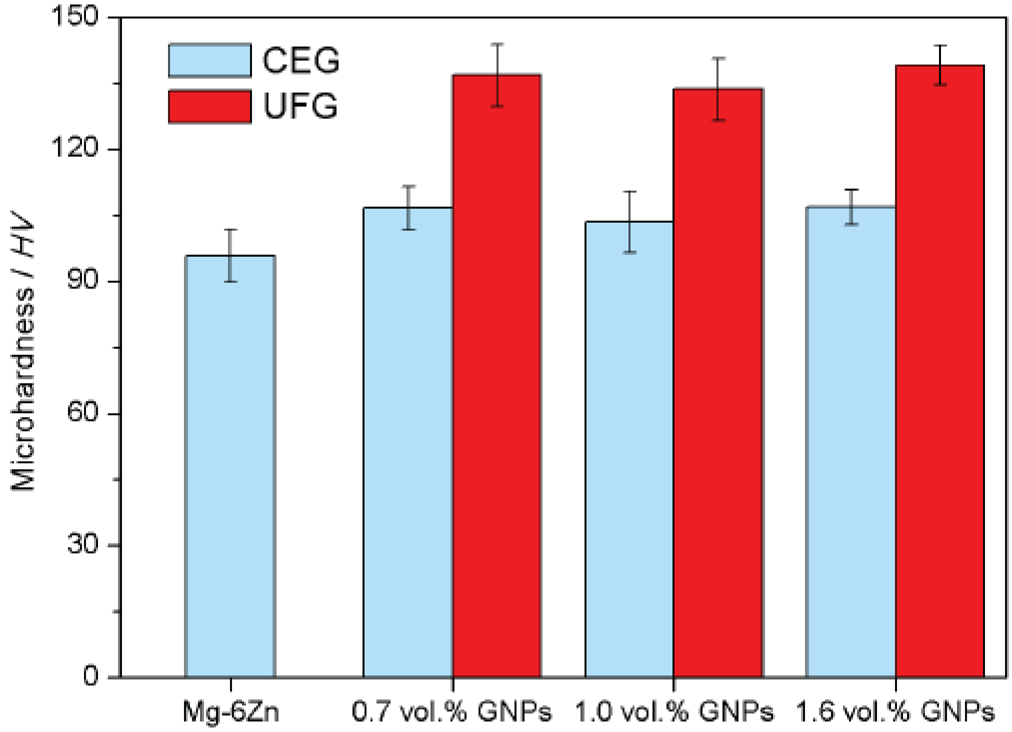

Figure S4. Vickers microhardness (*HV*) of the CEG and UFG lamellae in the composites and pure alloy. Error bars represent s.d. of six data sets.

Supplementary information 5

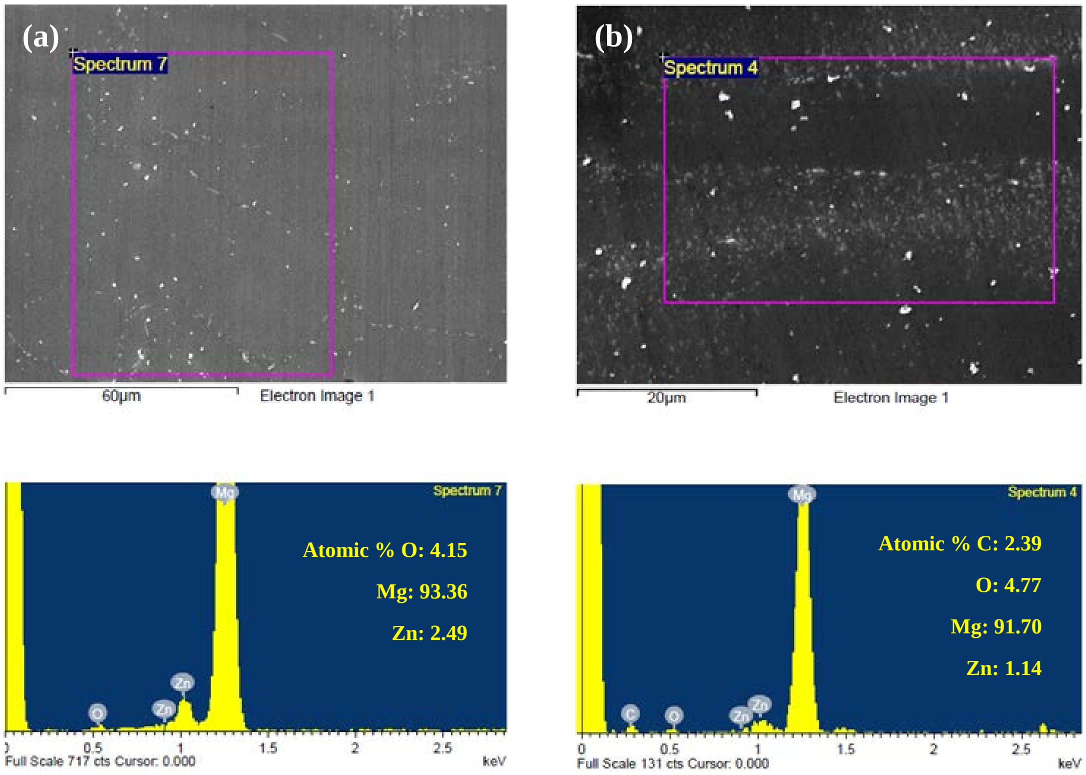

Figure S5. EDS analysis of the composition of (**a**) Mg-6Zn alloy and (**b**) 1.6 vol.% GNPs reinforced composite.
